# Effect of Age and Breed on Carcass and Meat Quality Characteristics of Beni-Guil and Ouled-Djellal Sheep Breeds

**DOI:** 10.1155/2021/5536793

**Published:** 2021-05-18

**Authors:** Kamal Belhaj, Farid Mansouri, Aziz Tikent, Yassin Taaifi, Mohamed Boukharta, Hana Caid Serghini, Ahmed Elamrani

**Affiliations:** ^1^Laboratory of Agricultural Production Improvement, Biotechnology and Environment, Faculty of Sciences, University Mohammed First, 717, Oujda 60000, Morocco; ^2^Veterinary Service, National Office for Food Safety, BP 73, Oujda 60000, Morocco; ^3^Institute of Agricultural Industries, High School of Charlemagne, Huy 4500, Belgium

## Abstract

Two hundred eight sheep, belonging to two main breeds of eastern Morocco, “Beni-Guil” and “Ouled-Djellal,” were investigated for carcass traits and meat quality. The objective of this study is to determine the effects of breed and age at slaughter on carcass traits and meat quality. The animals were slaughtered at three age classes: milk teeth, animals with two adult teeth, and adult animals. Dressing percentage, carcass measurements, compactness indices, carcass shrinkage parameter, conformation scores, fatness state, fat consistency, fat and meat colour, and pH were measured 24 hours postmortem. The results showed that the dressing percentage, carcass measurements, and compactness indices increased with slaughter age (*p* < 0.001). Furthermore, the effect of breed was higher for Ouled-Djellal breed of carcass characteristics (*p* < 0.01). However, no significant effect was observed for carcass shrinkage parameter. The *longissimus lumborum* muscle was used to determine the meat colour, which ranged from 23.89 to 21.96, while the ultimate pH ranged from 5.70 to 5.82. These results were influenced by age at slaughter (*p* < 0.05) but not influenced by breed. Breed and age at slaughter had a significant influence on carcass traits (*p* < 0.001). The present study provided a new insight into the effect of breed and age at slaughter on carcass and meat traits of both “Beni-Guil-PGI” and Ouled-Djellal sheep breeds.

## 1. Introduction

Despite the deteriorating state of the eastern Morocco highlands, their grazing areas contribute largely to feeding its livestock. The Beni-Guil (BG) breed (also called “Daghma” or “Hamra”) remains the dominant sheep breed in the oriental with its particular specificities; it is an autochthonous breed by transhumance, highly widespread for its rusticity, adaptability, prolificacy, performance in fattening, and industrial crossbreeding. Since 1980, the Beni-Guil breed was registered in the national sheep program of genetic improvement called “Plan Moutonnier.” This national plan aimed to improve and preserve autochthonous breeds, and the organization of farmers in cooperatives, and ensure technical support of national association of sheep and goat (ANOC: Association Nationale des Ovins et Caprins). It is noted that this program is based uniquely on phenotypic traits (head and legs colour, body weight, conformation, height at withers), as an animal breeding mass selection method that did not take into account carcass distinctive features. Hence, characters related to carcass quality remain unfortunately ignored. Since 2011, the IMANOR (Marocain institute of Normalisation) has granted a Protected Geographical Indication (PGI) to Beni-Guil lambs from eastern Morocco. These autochthonous breeds (BG and ODj) are the main source of red meat and income for local farmers. Most of them exploit the Beni-Guil sheep breed as selection flocks subsidized and supported by the government, and the Ouled-Djellal sheep breed as commercial flocks to meet the needs. Nevertheless, little information is available about traditional meat production systems meat production in eastern Morocco.

Sheep carcass is a major indicator of its marketability and economic value. It is determined by several criteria which are mainly the conformation, fatness state, dressing (yield), meat colour, fat cover quality (consistency and colour), and carcass linear measurements. The physiochemical traits of meat determine its commercial value and its acceptability by consumers [1]. The meat colour is the most important parameter quality for consumers. Generally, the meat of light and young lambs is more appreciated by consumers than that of heavier and adult animals especially due to its tenderness, flavour, and fat content [2]. Objectively, carcass description can be expressed by several direct and indirect measurements, which translates its quality and reflects its commercial value. Moreover, lambs' meat quality is influenced by several factors such as breed [3], slaughter age [4, 5], and weight at slaughter [2, 6]. We hypothesized that breed and age at slaughter have effects on the economic value of the sheep carcass of BG and ODj lamb meats. This study focused, on one hand, on determining the effect of age at slaughter on the objective and subjective characteristics of the BG breed carcass and meat to contribute to the characterization of this eastern Morocco label and, on the other hand, comparing carcass characteristics and meat quality between BG as an indigenous breed and ODj as a breed of Algerian origin.

## 2. Materials and Methods

### 2.1. Animals

Animals in eastern Morocco were reared on natural highlands in an arid to a semi-arid environment under the pastoral farming system. It is a system based on grazing throughout the year (8–12 months/year depending on rainfall availability) with certain supplementation. In this region, animals moved south in winter and north in summer within the same area to graze on the Alfa (*Stipa tenacissima*) and wormwood (*Artemisia herba-alba*) steppes. The dominant plant species in the steppe and highland pasture of Eastern Morocco included wormwood (*Artemisia herba-alba*), alfa grass (*Stipa tenacissima*), sparta grass (*Lygeum spartum*), Atriplex (*Atriplex halimus)*, ray-grass (*Lolium perenne* L.), laser white (*Laserpitium latifolium*), and sweet broom (*Arthrophytum scoparium*), along with the presence of other species, such as *Bromus* spp., *Eruca vesicaria* (roquette), *Stipa capensis*, and *Medicago* spp. The diet of the ewes was composed of natural pastures when available mainly based on alfa and wormwood formation with the presence other species depending the season. According to rainfall availability, some concentrate-based supplementation (200 to 250 g/day) is distributed to ewe based on alfalfa hay and barley in drought, food hunger season (connecting periods) and in some physiological stages, such as at the time of preparation for breeding (flushing) and for lambing (steaming). The lambing season occurred mainly from September to December. Lambing percentage was 95% and the mortality rate from birth to weaning is 2% for the tow-studied breeds. Four weeks before parturition, ewes received barley supplementation to avoid abortions, as well as to improve milk quality and production. Fifteen days after lambing, the lambs were vaccinated against enterotoxaemia and treated against internal and external parasites. In the first two months of age, the lambs stayed in the sheepfold during the day, giving them a supplement based on barley and alfalfa hay, ad libitum. Then, the lambs were raised with their mothers until weaning at the age of 3 months and fed on natural grassland pastures with additional feed based on barley (100–120 g/day) and hay. Before slaughter, the lambs undergo a finishing phase of 45 days based on barley (1 to 1.5 kg/day). The lambs had free access to water and mineral supplement in the form of a lick block.

**Figure 1:**
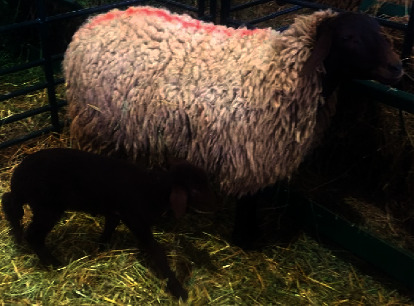
Ewe of Beni-Guil breed (18 months old) with its lamb.

**Figure 2:**
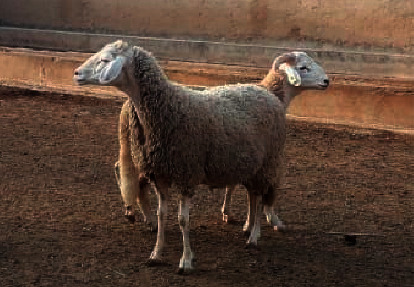
Ewe of Ouled-Djellal breed (18 months old) with its lamb.

At the waiting room of the slaughterhouse (in the ante-mortem inspection), the healthy animals have been randomly selected under the supervision of the veterinarians of the slaughterhouse based on the phenotypic characteristics of each breed:

- Beni-Guil breed (Figure 1): fire brown head, ears, prothorax, and legs without white spots; white fleece; absence of horns in the female; the head and the paws are devoid of wool. The rams usually have coarse and long horns that are pale and spiral. It is a breed characterized by its hardiness, its adaptability, its prolificacy, and its performance in fattening and industrial crossbreeding.
- Ouled-Djellal breed (Figure 2): it is an all-white breed (fleece, head, belly, and white limbs). In terms of the comparative assessment, the Ouled-Djellal breed is very common among breeders in the eastern region of Morocco by its growing characteristics, and therefore, it is profitable than that of Beni-Guil. Despite its importance, in terms of numbers (30% of the sheep population in the eastern region of Morocco) this breed is not involved in the national sheep program of genetic improvement. For this reason, the breeders operate alongside their state-subsidized Beni-Guil selection herds and commercial herds based on Ouled-Djellal to meet the various needs.

Two hundred and eight animals (female lambs and ewes) of the Beni-Guil (*n* = 90) and Ouled-Djellal (*n* = 118) sheep breed were included to study the effect of breed and age at slaughter on carcass characteristics and meat quality. The animals were divided into three groups for each breed according to a factorial schema 2 × 3 (two breeds—BG and ODj and three slaughter ages—A1 (*n* = 39 lambs for each breed) included animals from six months to one year (milk teeth); A2 (*n* = 34 lambs for each breed) included animals between one and two years (animals with two adult teeth); and A3 (*n* = 31 lambs for each breed) included animals between two and four years (adult animals)) [7]. The studied breeds were reared in the same system and production with the same pasture availability and quality. The animals were provided by the same breeders of Ain Beni-Mathar region who exploit the Ouled-Djellal breed alongside the Beni-Guil breed and supervised by the ANOC. The slaughtered lambs were all from simple birth. This work took place during the period between February and June 2017 in coordination with the veterinary service at two slaughterhouses: Oujda (34°41′12″ North, 1°54′41″ West) and Jerrada (34°18′42″ North, 2°09′49″ West). The animals underwent a resting period under a water diet for 16–24 h before slaughter, and the slaughter procedure was carried out according to the Halal procedure. The carcasses were refrigerated in cold rooms for 24 h at 4 ± 2°C.

### 2.2. Carcass Measurements

Postmortem, hot carcass weight (HCW) was recorded 45 min after slaughter. Carcass temperature was measured in the *longissimus lumborum* muscle as the reference muscle (LLM) to verify the temperature homogeneity of the samples used in this study. Three measurements were made for each sample and the mean temperature was calculated. Then, the carcass was kept in ambient temperature for 2–4 h to avoid cryo-choc [1]. Cold carcass weight (CCW) was recorded 24 h after the slaughter, and the hot and cold dressing were calculated using the live weight (HD = (HCW/LW^*∗*^100) and CD = (CCW/LW^*∗*^100), respectively, for hot dressing and cold dressing). Shrinkage loss (SL) was calculated as the difference between HCW and CCW (SL% = (HCW − CCW)^*∗*^100/HCW) according to Carrasco, Ripoll [8].

The carcasses were evaluated under the supervision of a veterinarian and using the notation scales as suggested and proposed in [8] and shown as follows:

- Fatness scale (1 = very low; 2 = low; 3 = average; 4 = high; and 5 = very high)
- Conformation scale (E excellent = 5; U very good = 4; R good = 3; O fair = 2; and P poor = 1)
- Cover fat quality scales (fat colour scale: 1 = very white; 2 = slightly coloured; 3 = partially coloured; 4 = strongly coloured)
- Fat firmness scale (1 = hard; 2 = firm; 3 = soft; 4 = very soft and oily)

Objective zoometric measurements were carried out according to literature [9]. The leg and carcass compactness index was calculated as the ratio between basin width/length leg and between CCW/carcass length, respectively [8, 10].

### 2.3. Meat Quality Measurements

The pH was measured 24 hours after slaughter in a laboratory using a pH meter (RADWAG, WPS510/C/2). We have taken 2 g of the LLM previously crushed in a grinder (chopper to Moulinex type knives) for 10 s. Then, the sample was homogenised in a homogeniser (WisdStir-MSH20D) for 10 to 15 min with 20 ml of distilled water and then filtered. The measurements were done on the supernatant in triplicate. The meat colour was measured 24 h post mortem on fresh surface of carcass, precisely in LLM (between 11^th^ and 13^th^ ribs) in three readings for each carcass with a Minolta CR300 (Minolta corporation, Ramsey, NJ, USA) according to the international centre of lighting using CIELAB system (L^*∗*^ = clarity, a^*∗*^ = red-green colour, b^*∗*^ = yellow-blue) [11]. (C^*∗*^ = (a^*∗*^^2^ + b^*∗*^^2^)^1/2^) and hue angle (H^*∗*^ = arctan (b^*∗*^/a^*∗*^)) were calculated to get an idea about the variation, concentration, and the chemical status of myoglobin, according to the breed, slaughter LW, and animal's age at slaughter [12].

### 2.4. Statistical Analysis

Statistical analyses were conducted using Statistical Package for the Social Sciences (IBM SPSS. 20). The normal distribution was verified according to the Shapiro–Wilk test for quantitative variables. A two-way analysis of variance (ANOVA) was carried out for the animal's age at slaughter (A), breed, and interaction between breed and age. Gabriel's post hoc test was used for means comparison. The difference was considered significant at *p* < 0.05. Principal component analysis (PCA) was performed on the data set to be sure whether it was possible to differentiate the samples according to their breed and age at slaughter and to obtain more information on the variables that mainly influence the carcass of our samples.

## 3. Results and Discussion

The effects of breed and animal's age at slaughter on carcass traits (linear and weight measurements) and meat quality of the two most breeds from eastern Morocco were considered and registered. The main results are reported in Tables 1–3.

### 3.1. Weight Measurements of Carcass

The weight measurements (carcass yield) were the main economic indicators and selection criteria in animal husbandry [13]. Except for shrinkage parameter, weight measurements of BG and ODj breeds at slaughterhouses showed significant increase (*p* < 0.01) in the weight and dressing percentage (Tables 1 and 3).

These findings were comparable to those reported by Alexandre, Bocage [14], Polidori, Pucciarelli [15] and Budimir, Trombetta [16] in the carcass of male lambs of other breeds. In addition, the breed had a significant effect on these parameters, where the higher values were recorded for ODj breed (*p* < 0.01). Similar results were reported by Santos-Silva and Mendes [17, 18]. However, Mateo and Caro [19] did not report any variation in dressing percentage for Assaf and Churra male lambs. On the other hand, no interaction between age and breed effects on weight measurements was recorded (Table 3).

The hot and cold carcass dressings were significantly influenced (*p* < 0.001) by age (Tables 1 and 2). In agreement with the previous studies [5, 15], increased slaughter age significantly increased the dressing percentages. In contrast, Budimir and Trombetta [16] reported a negative correlation between age and dressing percentage for male lamb slaughtered at 60 and 90 days.

This correlation could be attributed to offals weight and gastro intestinal content of heavier lambs, which are negatively correlated with dressing percentage. These data could be due to the different slaughter weights used in these two studies and by the rumination phenomena (functional as ruminants or not). In the present study, the older animals were completely functional as ruminants comparing to younger animals, which affect the dressing percentage. The recorded carcass yields were higher than those reported by Yousefi et al. [20] for Chall and Zel lambs and were comparable to those reported for Oula male lambs by Liu et al. [2]. However, the dressing percentages of the lambs from three age groups were lower than those reported for Apulian and Leccese male lambs slaughtered at 45 and 90 days of age [4, 21], respectively. For both breeds (BG and ODj), the lower dressing percentages were found at Age 2 (Table 1), and this result could be explained by the puberty age of animals and effects related to their sexual activity.

Shrinkage was due to cooling loss of carcass during the transformation of muscle into meat in the cold chamber. The meat market demand well-muscled carcasses with a high percentage of lean meat and a certain degree of fatness to prevent weight loss during the cold storage [22]. In this study, no perceivable significant effects of breed and age on the shrinkage loss were detected (*p* > 0.05). The higher carcass values (shrinkage loss) were recorded in younger lambs for both breeds. This result could be attributed to the fatness state (finishing degree) of animals used in this study of each group since the fat layer is an obstacle that prevents water loss from the carcass [23]. Our findings were lower than those reported by D'Alessandro, Maiorano [4] for male lambs slaughtered in spring and for female and male of Florida suckling kids slaughtered at different weights [22]. Finally, according to weight measurements results (Table 1), ODj sheep carcasses have higher weight yield than BG breed carcasses.

### 3.2. Linear Measurements of Carcass

Carcass linear measurements (K, G, and F) and carcass indexes (CCI 1, CCI 2, and LCI) are important criteria to predict carcass conformity often used by professionals to foresee its commercial value [9]. The BG and ODj carcass measurements (Table 1) revealed that K, G, and F increased significantly (*p* < 0.001; Table 3) with age at slaughter. Thus, carcass compactness indexes of both breeds were positively correlated with age at slaughter (*p* < 0.01). Similar correlations were reported in Cordeiro Mirandês female lambs [10], in Cornigliese female lambs [5], and in Fabrianese male lambs [15]. However, compactness indices are not affected by breed effect (*p* > 0.05). These indices have a significant influence only on carcass linear measurements. Santos-Silva and Mendes [17] have reported a similar breed effect on carcass linear measurements. However, Mateo and Caro [19] reported a significant effect of breed on compactness indices in Churra and Assaf Spanish sheep breed. Moreover, no significant interaction (*p* > 0.05) between studied factors was observed. The higher values of carcass measurements were recorded within the age-class 3 (A3) for ODj breed. This latter is the most profitable breed for breeders in the Oriental region of Morocco.

### 3.3. Carcass Conformation and Fatness

The results showed that the conformity and fatness score of BG and ODj sheep carcass (Tables 1, 3, and 4) were affected positively and significantly by age (*p* < 0.001); however the interaction between studied factors was not significant. The lowest values were recorded in younger lambs of both studied breeds. Similar results were found in Manchego sheep breed (Dıaz et al. [24]), in male of Rasa Aragonesa, Churra, and Merino Spanish breeds (Martínez-Cerezo et al. [1]), and in male of Martinik French sheep breed (Alexandre, Bocage) [14]. The fat cover firmness (fat consistency) was unaffected by these parameters (*p* > 0.05) in both breeds. Nevertheless, in the ODj breed, the carcass cover fat becomes gradually coloured with age at slaughter for age-class (Table 1).

This result showed that the breed had an impact on the subjective classification of carcasses. The higher values of conformity were recorded for BG sheep carcasses, but the higher fatness scores were recorded for ODj sheep carcasses. In conclusion, BG sheep carcasses showed better subjective characteristics in terms of conformity, fattening status, and fat cover firmness, and therefore carcasses sought by consumers.

### 3.4. Meat Quality

#### 3.4.1. Ultimate pH (pHu)

Meat pH is an important physicochemical quality parameter used to assess meat quality [12, 25]. In meat, the normal ultimate pH (pHu) value is around 5.60; a pHu value higher than 5.90 is undesirable [2]. The *longissimus Lumborum* muscle is used to assess meat quality parameters (pH_24_ and meat colour). The recorded values of pH_24_ of the samples ranged from 5.70 to 5.82 (Tables 2 and 3). The pH values measured in this study were accepted for commercial meats and were similar to values reported by D'Alessandro, Maiorano [4] for Lecesse lambs slaughtered in spring (5.77–5.82) and by Liu et al. [2] for Oula lamb (5.80–5.83). However, these recorded values were higher than those displayed by certain Turkish breeds slaughtered at different slaughter weights (5.50–5.67) [26]. The results showed a negative correlation between pH_24_ values and the age at slaughter (*p* < 0.05), whereas the breed did not affect the ultimate pH value. This negative correlation was due to the level of glycogen reserves between young and adult lambs. Adults absorbed greater amount of carbohydrates (mainly cereals) compared to younger lambs; as a consequence, ruminal fermentation is promoted and thus the production of propionic acid, the precursor of muscle glycogen [27]. The results reported in literature on the effect of slaughter weight on the pH value were variable. Several studies found a significant effects of slaughter weight on ultimate pH for sheep meat, with higher pH in heavy lambs, possibly because older lambs (greater live weight at slaughter) may be more sensitive to pre-slaughter stress [2, 28] and are in line with our results. However, numerous workers did not find any effects of slaughter weight on ultimate pH for sheep meat [1, 10, 29, 30].

#### 3.4.2. Meat Colour Parameters

Consumer preferences for meat colour and packaging influence likelihood to purchase. Nonetheless, meat colour remains the main visual parameter assessed by consumers and the most impacting criterion in the meat purchase decision [12, 25, 31]. Indeed, it is an essential parameter in meat industry, in which carcasses should be pink or pale pink, preferred by the consumers, while darker meat is considerably difficult to market [2, 32].

In the present study, the lightness values ranging between 40.89 and 41–89 for all the groups are indicative of bright-red meat [10]. In line with previous studies [2, 33], the slaughter age influenced significantly the meat colour (Table 2). Generally, the lightness was decreasing with increasing age at slaughter, and the redness increased, while yellowness (b^*∗*^) did not appear to be influenced by age. Chroma (C^*∗*^) was significantly different (*p* < 0.001) between the three groups of animals; however hue angle (H^*∗*^) did not show significant differences between the two groups of animals (Table 3). These variations in meat colour of BG and ODj sheep according to slaughter age can be explained by the increase of iron and myoglobin (heme pigment) concentration with age, likewise for the ultimate pH values recorded in each age class [34, 35], whereas the breed does not affect the meat colour parameters (*p* > 0.05), and no interaction effect between breed and age was recorded (*p* > 0.05).

As shown in Table 2, for young animals (A1), meat colour was bright-red, while in adult animals (A2 and A3) it changed from bright-red to red. Similar results were reported in Rasa Aragonesa, Churra, Merino, and Assaf sheep breeds [1] (in Leccese breed [4], and in Pantaneiro Brazilian breed [35]. In contrast, a significant breed effect on meat colour for two Canarias Island breeds (Canaria and Canaria Hair) was recorded [6]. No breed effect was observed in this study for BG and ODj sheep (*p* > 0.05). It is a predicted result since the two studied breeds (BG and ODj) were reared in the same conditions under traditional livestock system.

### 3.5. Principal Component Analysis of the Carcass and Meat Quality Characteristics

**Figure 3:**
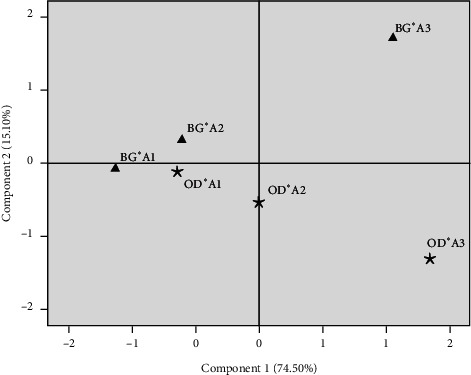
Two-dimensional principal component analysis plot for carcass traits, of Beni-Guil and Ouled-Djellal sheep, according to animals' age (A) at slaughter; A1: six months to one year; A2: one to two years; A3: two to four years.

A principal component analysis (PCA) was performed to classify and determine the relationships between the studied animals, taking into account the combined effects of breed and slaughter age. The first component explained 74.50% and the second explained 15.10%.  Figure 3 provides the global representation of animals studied in the factorial plane consisting of PC1 and PC2. The individual projection on the factorial map showed discrimination between the studied animals making it possible to summarize the interpretations already mentioned above in a very simplified way, in terms of the evaluated parameters. Compared to PC1, the studied animals could be divided into three groups. The first consists of animals of BG and ODj breeds slaughtered at A1 (animals of six months to one year), and BG animals slaughtered at A2 (between one and two years). The Ouled-Djellal breed slaughtered at A2 characterized the second group. Finally, the third group was composed of Beni-Guil and Ouled-Djellal breed slaughtered at A3 (between two and four years). Furthermore, these results showed the interaction effects of breed × age on the carcass objective characteristics.  Table 4 gives more gives more explanation to understand this classification. Actually, the first group is correlated with the parameters of the meat colour (hue, lightness, and redness) and ultimate pH. It indicated that animals slaughtered at A1 and those of BG slaughtered at A2 had an optimal red coloration (bright-red colour). The third group, which is the opposite of the first group with respect to PC1, was characterized by a positive correlation with carcass compactness (weight and linear measurements of carcass) and redness index, showing that animals slaughtered at A3 had a redder meat colour and a low pH value compared to groups 1 and 2.

For principal component 2, the most distinct variables were shrinkage loss, yellowness, and carcass compactness index 1 (Table 4). Considering the PC2, the breed effect has mainly manifested. As shown in Figure 3, the animals of the ODj breed slaughtered at A3 class are opposite to those of BG-A3. The latter is characterized by high values of carcass compactness index 1 (G/F), high shrinkage loss, and a redder colour. In addition, the results of this statistical analysis allow us to highlight a clear variability between studied animals according to data on carcass and meat quality characteristics. These results are in line with previous research carried out on lambs and revealed the similar effects from different geographical areas.

## 4. Conclusion

This is the first time a scientific-based study reports on carcass and meat quality characteristics of two main sheep breeds (Beni-Guil and Ouled-Djellal) reared in the highlands of eastern Morocco. The slaughter age could be used as a selection criterion by professionals and consumers to classify sheep carcass and meat in eastern Morocco. Generally, dressing percentage and carcass compactness indices increase with higher slaughter age, where the higher values of carcass weight were recorded in the Ouled-Djellal sheep breed carcass. Adult animals present the highest conformation score and fatness degree. The better conformation score was observed in Beni-Guil sheep breed carcass, while the highest fatness degree was observed in Ouled-Djellal sheep breed carcass. No breed differences were found between breeds for ultimate pH (pH24). To increase consumers' loyalty, the PGI label should be taken into account in their regulatory directive a range of slaughter age, to provide consumers a homogeneous and standard product from one purchase to another. Further investigations are needed for more comprehensive understanding of age at slaughter effects on meat and carcass quality characteristics, evaluating meat yield, nutritional and sensorial quality, and to study the season and feed effects.

## Figures and Tables

**Table 1:** Effect of breed and age at slaughter on carcass traits of Beni-Guil and Ouled-Djellal sheep breed from eastern Morocco (means ± SD).

Parameters	Beni-Guil (BG)			Ouled-Djellal (ODj)		
	Age 1 (*n* = 30)	Age 2 (*n* = 30)	Age 3 (*n* = 30)	Age 1 (*n* = 39)	Age 2 (*n* = 39)	Age 3 (*n* = 40)
*Weight measurements*						
SLW (kg)	22.91 ± 2.10^a^	32.21 ± 2.10^b^	45.10 ± 2.00^c^	24.88 ± 3.23^a^	36.15 ± 3.68^d^	47.54 ± 1.52^e^
HCW (kg)	11.12 ± 1.30^a^	15.25 ± 1.16^b^	22.41 ± 1.23^c^	12.35 ± 1.64^a^	17.43 ± 1.59^d^	24.79 ± 1.57^e^
CCW (kg)	10.90 ± 1.28^a^	14.97 ± 1.14^b^	21.96 ± 1.17^c^	12.14 ± 1.66^a^	17.12 ± 1.57^d^	24.41 ± 1.53^e^
HD (%)	48.53 ± 0.01^ab^	47.34 ± 0.01^a^	49.68 ± 0.01^b^	49.63 ± 0.02^ab^	48.21 ± 0.01^ab^	52.14 ± 0.02^c^
CD (%)	47.57 ± 0.01^ab^	46.47 ± 0.01^a^	48.69 ± 0.01^b^	48.79 ± 0.02^ab^	47.35 ± 0.01^ab^	51.34 ± 0.02^c^
SL (%)	1.97 ± 0.40	1.83 ± 0.36	2.00 ± 1.37	1.70 ± 0.49	1.75 ± 0.46	1.53 ± 0.72

*Linear measurements*						
G (cm)	15.24 ± 1.47^a^	16.64 ± 0.61^b^	20.60 ± 1.49^c^	15.98 ± 1.22^ab^	17.90 ± 1.15^d^	20.09 ± 1.32^c^
K (cm)	58.27 ± 5.63^a^	61.49 ± 0.58^b^	73.01 ± 1.45^c^	61.67 ± 2.48^b^	66.66 ± 3.00^d^	76.87 ± 3.40^e^
F (cm)	27.93 ± 1.00^a^	31.65 ± 1.14^b^	32.78 ± 1.21^bc^	31.64 ± 1.51^b^	32.68 ± 1.84^b^	34.18 ± 2.54^c^
LCI = G/F	0.55 ± 0.04^a^	0.53 ± 0.03^a^	0.63 ± 0.06^c^	0.55 ± 0.04^ab^	0.54 ± 0.03^ab^	0.59 ± 0.05^bc^
CCI1 = G/K	0.26 ± 0.03^a^	0.27 ± 0.10^a^	0.28 ± 0.02^b^	0.26 ± 0.02^ab^	0.27 ± 0.01^ab^	0.26 ± 0.01^a^
CCI2 = CCW/K (g/cm)	187.06 ± 24.77^a^	243.45 ± 19.89^b^	300.78 ± 21.57^c^	296.85 ± 28.56^b^	256.82 ± 23.68^b^	317.54 ± 35.68^c^

*Subjective characteristics*						
EUROP-conformation	2.15 ± 0.37^a^	2.19 ± 0.40^a^	2.75 ± 0.44^bc^	2.47 ± 0.5^ab^	2.42 ± 0.5^ab^	2.91 ± 0.28^c^
EUROP-fatness	2.95 ± 0.20^a^	3.42 ± 0.50^b^	4.00 ± 0.00^c^	3.25 ± 0.44^ab^	4.09 ± 0.3^cd^	4.36 ± 0.49^d^
Fat colour	1.00 ± 0.00^a^	1.00 ± 0.00^a^	1.00 ± 0.00^a^	1 ± 0.00^a^	1.19 ± 0.40^ab^	1.33 ± 0.48^b^
Fat firmness	1.00 ± 0.00	1.00 ± 0.00	1.00 ± 0.00	1.00 ± 0.00	1.00 ± 0.00	1.00 ± 0.00

^a,b,c,d,e^Values with different superscript letters are significantly (*p* < 0.05) different within row. Age 1 = six months to one year; age 2 = between one and two years; age 3 = between two and four years; SLW: slaughter live weight; HCW: hot carcass weight; CCW: cold carcass weight; HD: hot dressing; CD: cold dressing; SL: shrinkage loss; G: basin width; K: carcass length; F: leg length. LCI: leg compactness index; CCI1: carcass compactness index 1; CCI2: carcass compactness index 2; EUROP-conformation: *E* = 5. *U* = 4. *R* = 3. O = 2. *P* = 1 (1 = poor 5 = excellent); EUROP-fatness: 1 = very low; 2 = low; 3 average; 4 = high; 5 = very high; fat colour scale: 1 = very white, 2 = slightly colored 3 = partially colored, 4 = strongly colored; fat firmness scale: 1 = hard, 2 = firm, 3 = soft, 4 = very soft and oily.

**Table 2:** Effect of breed and animal's age at slaughter on ultimate pH and meat colour of Beni-Guil and Ouled-Djellal sheep breeds from eastern Morocco (means ± SD).

Parameters	Beni-Guil (BG)			Ouled-Djellal (ODj)		
	Age 1 (*n* = 30)	Age 2 (*n* = 30)	Age 3 (*n* = 30)	Age 1 (*n* = 39)	Age 2 (*n* = 39)	Age 3 (*n* = 40)
pH ultimate	5.82 ± 0.05^a^	5.77 ± 0.08^ab^	5.70 ± 0.07^b^	5.81 ± 0.07^ab^	5.75 ± 0.02^ab^	5.72 ± 0.07^ab^
L (lightness)	41.59 ± 0.42	41.89 ± 0.18	40.89 ± 0.88	41.2 ± 0.59	41.36 ± 0.50	40.89 ± 0.88
a ^*∗*^ (redness)	20.95 ± 0.85^a^	21.11 ± 0.42^a^	22.73 ± 0.83^c^	20.81 ± 0.75^a^	21.3 ± 0.69^ab^	22.28 ± 0.48^bc^
b ^*∗*^ (yellowness)	7.04 ± 1.05	7.07 ± 0.69	7.37 ± 0.46	7.04 ± 1.05	6.81 ± 0.47	6.96 ± 0.71
Chromaticity	22.10 ± 1.05^ab^	22.26 ± 0.54^ab^	23.89 ± 0.81^c^	21.96 ± 0.81^a^	22.36 ± 0.62^ab^	23.34 ± 0.51^bc^
Hue angle°	18.57 ± 1.20	18.51 ± 1.56	17.96 ± 2.25	18.68 ± 2.67	17.72 ± 1.43	17.34 ± 1.70
a ^*∗*^/b ^*∗*^	2.97 ± 0.41	2.99 ± 0.27	3.08 ± 0.21	2.95 ± 0.49	3.12 ± 0.25	3.20 ± 0.36

^a, b, c^Values with different superscript letters are significantly (*p* < 0.05) different within row. Scales of L ^*∗*^, a ^*∗*^, b ^*∗*^, chroma, and Hue: a ^*∗*^ and b ^*∗*^: −60 to 60; L ^*∗*^: 0 to 100; chromaticity: 0–60; hue: 0–360°; A1: six months to one year; A2: between one and two years; A3: between two and four years.

**Table 3:** Effect of breed, slaughter age, and their interaction on carcass traits and meat quality of Beni-Guil and Ouled-Djellal sheep breed from eastern Morocco.

Parameters	Effect		
	Age	Breed	Age × breed
*Weight measurements*			
SLW (kg)	^*∗∗∗*^	^*∗∗∗*^	NS
HCW (kg)	^*∗∗∗*^	^*∗∗∗*^	NS
CCW (kg)	^*∗∗∗*^	^*∗∗∗*^	NS
HD (%)	^*∗∗∗*^	^*∗∗*^	NS
CD (%)	^*∗∗∗*^	^*∗∗*^	NS
SL (%)	NS	NS	NS

*Linear measurements*			
G (cm)	^*∗∗∗*^	NS	NS
K (cm)	^*∗∗∗*^	^*∗∗∗*^	NS
F (cm)	^*∗∗∗*^	^*∗∗∗*^	NS
LCI = G/F	^*∗∗∗*^	^*∗∗∗*^	NS
CCI1 = G/K	NS	NS	NS
CCI2 = CCW/K(g/cm)		NS	NS

*Subjective characteristics*			
EUROP-conformation	^*∗∗∗*^	^*∗∗∗*^	NS
EUROP-fatness	^*∗∗∗*^	^*∗∗∗*^	NS
Fat colour	^*∗*^	^*∗∗∗*^	NS
Fat firmness	NS	NS	NS

*pH and color parameters*			
pH ultimate	^*∗∗*^	NS	NS
L ^*∗*^ (lightness)	^*∗*^	NS	NS
a ^*∗*^ (redness)	^*∗∗∗*^	NS	NS
B ^*∗*^ (yellowness)	NS	NS	NS
Chromaticity	^*∗∗∗*^	NS	NS
Hue angle°	NS	NS	NS
a ^*∗*^/b ^*∗*^	NS	NS	NS

NS: not significant (*p* > 0.05); ^*∗*^*p* < 0.05; ^*∗∗*^*p* < 0.01; ^*∗∗∗*^*p* < 0.001; SLW: slaughter live weight; HCW: hot carcass weight; CCW: cold carcass weight; HD: hot dressing; CD: cold dressing; SL: shrinkage loss; G: basin width; K: carcass length; F: leg length; LCI: leg compactness index; CCI1: carcass compactness index 1; CCI2: carcass compactness index 2.

**Table 4:** Two main components explaining more than 83% of the total information on the carcass and meat quality traits of Beni-Guil and Ouled-Djellal sheep breeds.

Variables	Principal component		Variables	Principal component	
	1	2		1	2
SLW	0.973	0.028	HD	0.817	−0.343
HCW	0.986	−0.027	CD	0.818	−0.373
CCW	0.986	−0.033	pH ultimate	−0.934	−0.238
K	0.997	−0.080	L ^*∗*^	−0.842	−0.014
G	0.982	0.160	a ^*∗*^	0.941	0.259
CCI1	0.397	0.802	b ^*∗*^	0.123	0.929
LCI	0.824	0.408	Chroma	0.920	0.320
CCI2	0.915	−0.018	Hue	−0.853	0.442
SL	−0.427	0.788	a ^*∗*^/b ^*∗*^	0.817	−0.544

PC: principal component; SLW: slaughter live weight; HCW: hot carcass weight; CCW: cold carcass weight; HD: hot dressing; CD: cold dressing; SL: shrinkage loss; G: basin width; K: carcass length; F: leg length; LCI: leg compactness index; CCI1: carcass compactness index 1; CCI2: carcass compactness index 2.

## Data Availability

The data used to support the findings of this study are available from the corresponding author upon request.
